# IMMUNEPOTENT CRP increases intracellular calcium through ER-calcium channels, leading to ROS production and cell death in breast cancer and leukemic cell lines

**DOI:** 10.17179/excli2022-5568

**Published:** 2023-03-16

**Authors:** Helen Y. Lorenzo-Anota, Alejandra Reyes-Ruiz, Kenny M. Calvillo-Rodríguez, Rodolfo Mendoza-Reveles, Andrea P. Urdaneta-Peinado, Karla M. Alvarez-Valadez, Ana Carolina Martínez-Torres, Cristina Rodríguez-Padilla

**Affiliations:** 1 Laboratorio de Inmunología y Virología, Facultad de Ciencias Biológicas, Universidad Autónoma de Nuevo León, San Nicolás de los Garza, México; 2 Tecnológico de Monterrey, The Institute for Obesity Research, Monterrey, México; 3 LONGEVEDEN S.A. de C.V.

**Keywords:** calcium, ryanodine receptor, IP3 receptor, immunotherapy, cell death, cancer

## Abstract

IMMUNEPOTENT CRP (ICRP) is an immunotherapy that induces cell death in cancer cell lines. However, the molecular mechanisms of death are not completely elucidated. Here, we evaluated the implication of intracellular Ca^2+^ augmentation in the cell death induced by ICRP on T-ALL and breast cancer cell lines. Cell death induction and the molecular characteristics of cell death were evaluated in T-ALL and breast cancer cell lines by assessing autophagosome formation, ROS production, loss of mitochondrial membrane potential, ER stress and intracellular Ca^2+ ^levels. We assessed the involvement of extracellular Ca^2+^, and the implication of the ER-receptors, IP_3_R and RyR, in the cell death induced by ICRP, by using an extracellular calcium chelator and pharmacological inhibitors. Our results show that ICRP increases intracellular Ca^2+^ levels as the first step of the cell death mechanism that provokes ROS production and loss of mitochondrial membrane potential. In addition, blocking the IP_3_ and ryanodine receptors inhibited ER-Ca^2+ ^release, ROS production and ICRP-induced cell death. Taken together our results demonstrate that ICRP triggers intracellular Ca^2+^-increase leading to different regulated cell death modalities in T-ALL and breast cancer cell lines.

See also Figure 1(Fig. 1).

## Introduction

Despite the pivotal advances in the treatment of breast cancer and T-cell acute lymphoblastic leukemia (T-ALL), breast cancer is one of the leading causes of death among women (Siegel et al., 2019[37]), whereas T-ALL is the most diagnosed cancer in children worldwide (Karman and Johansson, 2017[16]; Terwilliger and Abdul-Hay, 2017[40]). One of the reasons is the ability of cancer cells to develop different mechanisms of resistance to the current therapies including cell death inductors. 

IMMUNEPOTENT CRP (ICRP) is a bovine dialyzable leukocyte extract (DLE) obtained from disrupted spleen; it has shown immunomodulatory properties and cytotoxic activity against cancer cell (Arnaudov, 2017[2]; Kirkpatrick 2000[19]). It is cytotoxic in solid cancer cell lines, including melanoma, cervical, lung and breast cancer (Martinez-Torres et al., 2018[24], 2019[22][23]; Reyes-Ruiz et al., 2021[33]; Rodríguez-Salazar et al., 2017[34]) and hematological malignances, such as T-ALL (Lorenzo-Anota et al., 2020[21]). Despite the molecular differences between the cancer cell linages evaluated, some characteristics of the ICRP-mediated cell death mechanism remain conserved among the different cancer types studied. In all cancer cell lines ICRP has demonstrated to enhance reactive oxygen species (ROS) production leading to loss of mitochondrial membrane potential, DNA damage that promotes cell cycle arrest and finally, DNA degradation (Lorenzo-Anota et al., 2020[21]; Martinez-Torres et al., 2018[24], 2019[22][23]; Reyes-Ruiz et al., 2021[33]). Interestingly, solid cancer cells exposed to ICRP succumb to a caspase-independent but ROS-dependent cell death, whereas this same agent induces caspase activation leading to ROS and caspase-dependent cell death in leukemic cells. 

We recently reported that ICRP induces characteristics related with endoplasmic reticulum (ER) stress such as autophagosome formation, translocation of ER chaperones to the cell surface, and eIF2a phosphorylation (P-eIF2a) (Almanza et al., 2019[1]) in cervical and breast cancer (Martínez-Torres et al., 2020[25]; Reyes-Ruiz et al., 2021[33]). Yet, these mechanisms have not been previously dilucidated in hematological malignancies. 

It has been shown that the depletion of the ER Ca^2+^ pool or ER-Ca^2+ ^overload results in disturbances that can lead to ER stress (Zhivotovsky and Orrenius, 2011[43]). As Ca^2+^ is a highly versatile second messenger, it regulates broad cellular functions, and disturbances in Ca^2+^ homeostasis can lead to cell death (Monteith et al., 2017[27]). Ca^2+^ alterations can lead to distinct types of regulated cell death mechanisms, such as caspase-dependent and caspase-independent cell death modalities, in different cancer types (Danese et al., 2021[10]). However, it is unknown if there are alterations in ER-Ca^2+^ homeostasis after I-CRP-treatment in solid or liquid cancers. To better understand the mechanism of action of ICRP, the purpose of this study was to evaluate for the first time the role of Ca^2+^ in the mechanism of cell death induced by ICRP in T-ALL and breast cancer cells. 

## Materials and Methods

### Cell culture

MCF-7 human breast adenocarcinoma (ATCC® HTB-22TM), 4T1 murine mammary adenocarcinoma (ATCC® CRL2539TM), and T-acute lymphoblastic leukemia cell lines CEM (ATCC® CCL-119™) and MOLT-4 (ATCC CRL-1582) were obtained from the American Type Culture Collection. MCF-7 cells were cultured in DMEM-F12 while 4T1, CEM and MOL-4 cells were cultured in RPMI-1640, all were supplemented with 10 % fetal bovine serum (FBS) and 1 % penicillin-streptomycin (Life Technologies, Grand Island, NY) and maintained in a humidified incubator in 5 % CO_2_ at 37 °C. Cell count was performed using 0.4 % trypan blue (MERCK, Darmstadt, Germany) in a Neubauer chamber.

### Cell death induction and inhibition 

IMMUNEPOTENT CRP (ICRP) was produced as previously described (Coronado-Cerda et al., 2016[9]; Franco-Molina et al., 2006[12]), and dissolved in cell culture media. One unit (U) of ICRP is defined as 24 mg of peptides obtained from 15×10^8^ leukocytes. We used different pharmacological inhibitors to determine the cell death mechanism of ICRP in cancer cell lines. QVD-OPh (QVD, 10 μM) as a general caspase inhibitor, N-acetylcysteine (NAC, 5 mM) was used as a ROS inhibitor, Spautin-1 (Sp-1, 15μM) as autophagosome inhibitor, BAPTA (50 μM) as extracellular calcium chelator, Dantrolene (30 μM) as ryanodine receptors (RyR) inhibitor, and 2-APB (30 μM) as inositol triphosphate receptor (IP_3_R) inhibitor. The inhibitors were added 30 minutes before ICRP (CC_50_) treatment. 

### Cell death analysis 

Cell death quantification was determined by analyzing phosphatidylserine exposure using annexin V-allophycocyanin (APC) (AnnV, 0.25 μg/mL; BD Biosciences Pharmingen, San Jose, CA, USA) and cell membrane permeability with propidium iodide (PI; 0.5 μg/mL; MilliporeSigma, Eugene, OR, USA) staining. In brief, 5×10^4^ cells were seeded and exposed to different concentrations of ICRP in subsequent assays; this allowed to define the median cytotoxic concentration of ICRP required to induce 50 % of cell death (CC_50_). After 24 h of treatment, cells were recollected and washed with phosphate-buffered saline (PBS), then resuspended in binding buffer (10 mM HEPES/ NaOH pH 7.4, 140 mM NaCl, 2.5 mM CaCl2), and stained during 20 minutes at 4 °C. Finally, cells were assessed in BD Accury C6 flow cytometer (Becton Dickinson, Franklin Lakes, NJ) and analyzed in FlowJo Software (LLC, Ashland, OR).

### Autophagosome formation assay

Autophagosome formation was assessed using Autophagy Detection Kit (Cyto-ID; Abcam, Cambridge, UK). In brief, 5×10^4^ breast cancer cells were cultured in 24-well plates (Life Sciences) and 1×10^5^ leukemia cells were cultured in 96-well plates (Life Sciences). Cells were treated with ICRP (CC_50_) for 24 h and then detached, washed with PBS, recovered, and stained following the manufacturer's instructions. Measurement was determined by flow cytometry and analyzed using Flowjo Software as mentioned previously.

### EIF2α phosphorylation assay

Breast cancer cells (5×10^4^) and T-acute lymphoblastic leukemic cells (1×10^6^) were plated in 6-well dishes (Life Sciences) and incubated with ICRP (CC_50_) for 18 h. Cells were then collected and fixed with methanol for 1 h at 4 °C, washed with 2 %-FACS Buffer (PBS 1× and 2 % FBS) and centrifuged twice at 1,800 rpm during 20 min. Next, cells were suspended in 50 μL of 10 %-FACS Buffer (PBS 1× and 10 % FBS), incubated for 30 min, and shaken at 400 rpm and 25 °C. After this, 0.5 μL of anti-EIF2S1 (phospho S51) antibody [E90] (Abcam, ab32157) was added, incubated for 2 h, and washed with 2 %-FACS Buffer. Cells were suspended in 100 μL of 10 %-FACS Buffer), incubated for 15 min, and shaken at 400 rpm and 25 °C, 0.5 μL of goat anti-rabbit IgG H&L (Alexa Fluor® 488) (Abcam, ab150077) was then added and incubated for 1 h in darkness. Cells were washed with 2 %-FACS Buffer and eIF2α phosphorylation was measured by flow cytometry, as mentioned before.

### Intracellular Ca^2+^ levels analysis

Intracellular calcium was assessed using Fluo-4 AM (Life Technologies). Breast cancer cells (5×10^4^) and T-acute lymphoblastic leukemic cells (1×10^6^) were plated in 6-well dishes (Life Sciences) and incubated with ICRP CC_50_ for 18 h. After treatment, cells were washed twice with KREBS buffer, resuspended in RINGER buffer with 0.001 μg/mL of Fluo-4 AM (Life Technologies) and 0.001 μg/mL of Pluronic F-127 (Life Technologies), and incubated at 37 °C for 30 min in darkness. Next, cells were washed twice with RINGER buffer. For fluorescence microscopy, cells were placed on microscopy slides with coverslips, assessed by confocal microscopy (OLYMPUS X70) and analyzed with Image-J software.

For intracellular calcium quantification cells were collected, washed, and assessed by flow cytometry and results were analyzed using FlowJo Software (LLC, Ashland, OR).

### Mitochondrial membrane potential assessment

To determine mitochondrial damage, we tested loss of mitochondrial membrane potential by tetramethylrhodamine ethyl ester stain (TMRE, 50 nM; Sigma, Aldrich, Darmstadt, Germany). In brief, 5×10^4^ cells were incubated with ICRP (CC_50_) for 24 h in presence or absence of 1.5 mM BAPTA (MERCK). Cells were then harvested, washed with PBS, stained, incubated at 37 °C for 30 min, and measured by flow cytometry as described above. 

### ROS generation analysis

ROS levels were determined by staining cells with 2′,7′-Dichlorofluorescin diacetate (DCFDA; 2.5 μM; MERCK). In brief, 5 × 10^4 ^cells/well were incubated with ICRP (CC_50_) during 24 h in presence or absence of BAPTA (1.5 mM; MERCK). Cells were then detached, washed with PBS, stained, incubated at 37 °C for 30 min, and measured using a flow cytometer, as mentioned before.

### Cleaved caspase-3 analysis

We used a specific detection kit, FITC-DEV-FMK (ABCAM; Cambridge, UK) to assess caspase-3 activation. In brief, 1×10^5 ^cells/well were incubated with ICRP (CC_50_) for 24 h, then cells were recovered and stained following the manufacturer's instructions. Results were measured using a flow cytometer, as mentioned before.

### Statistical analysis

Data were analyzed using GraphPad Prism Software (GraphPad Software Inc., San Diego, CA) and showed as mean ± SD of triplicates from three independent experiments. Statistical analyses were done using the paired Student's t-test. The statistical significance was defined as *p* < 0.05. 

## Results

### IMMUNEPOTENT-CRP triggers different ROS-dependent cell death modalities depending on the cancer cell lineage

**Figure 2:**
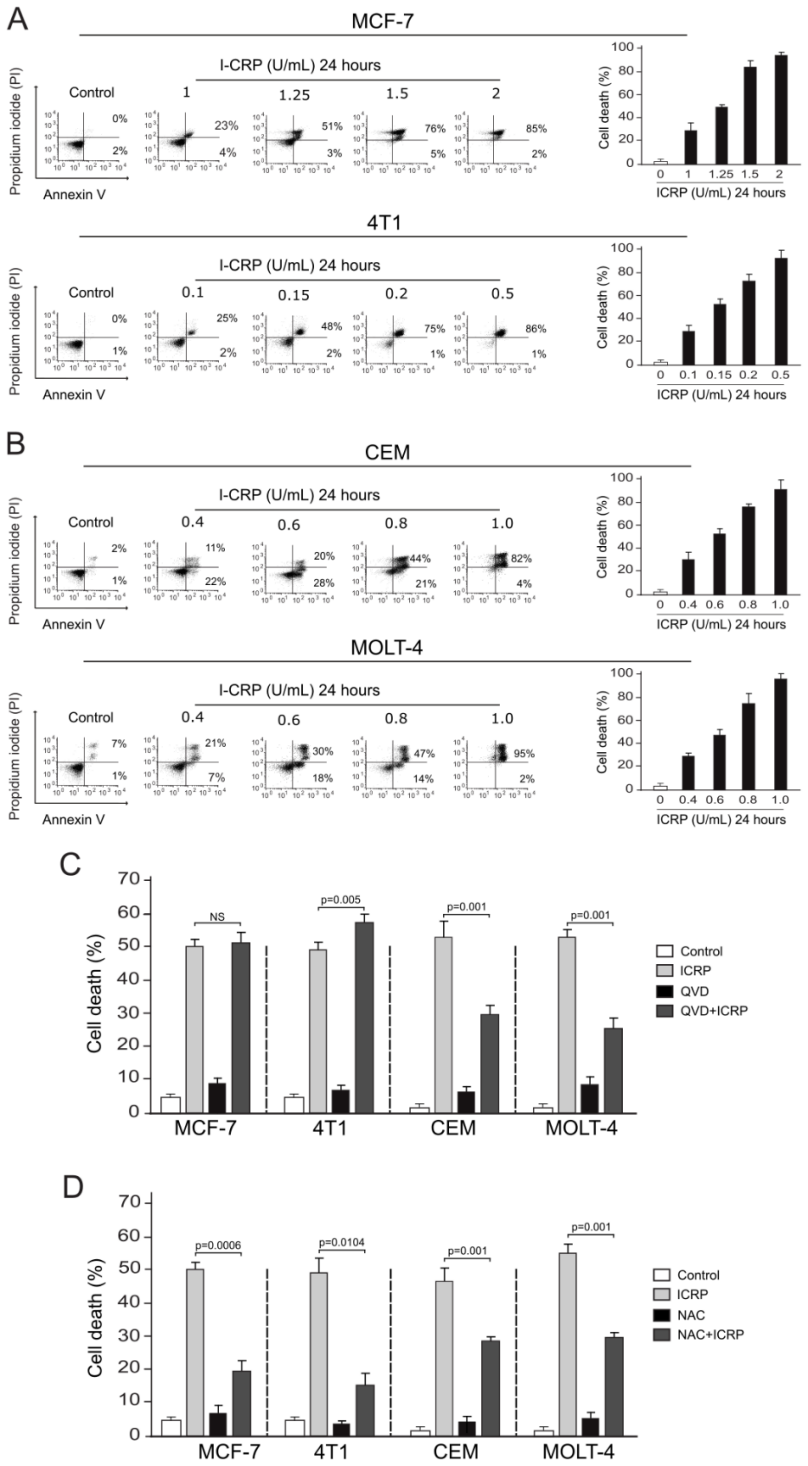
IMMUNEPOTENT CRP induces different regulated cell death modalities concentration and ROS-dependent manner on breast cancer and in T-ALL cell lines. A-B. Representative dot plots and quantification of cell death measured by flow cytometry using Annexin-V and PI staining in breast cancer cell lines MCF-7 (above) and 4T1 (down) (A) and in T-acute lymphoblastic leukemia cells lines CEM (above) and MOLT-4 (down) (B) treated with different concentrations of ICRP for 24 h. C. Cell death quantification of MCF-7, 4T1, CEM and MOLT-4 cell lines left alone or pretreated with a pan-caspase inhibitor QVD before ICRP CC_50_ treatment (24 h). D. Cell death quantification in MCF-7, 4T1, CEM and MOLT-4 cell lines left alone or pretreated with the antioxidant NAC before ICRP CC_50_ treatment (24 h). The means (± SD) of triplicates of at least three independent experiments were graphed.

ICRP is cytotoxic in a concentration-dependent manner on breast cancer (MCF-7 and 4T1) (Figure 2A(Fig. 2)) and T-ALL (CEM and MOLT-4) (Figure 2B(Fig. 2)) cell lines provoking phosphatidyl serine exposure and cell membrane permeabilization. Cell death in 30 % of cells (CC_30_) was reached at 1 U/mL, 50 % (CC_50_) at 1.25 U/mL, 80 % (CC_80_) at 1.5 U/mL, and 100 % (CC_100_) at 2 U/mL in MCF-7 cells (Figure 2A above(Fig. 2)). In 4T1 cells 30 % of cell death (CC_30_) was reached at 0.1 U/mL, 50 % (CC_50_) at 0.15 U/mL, 80 % (CC_80_) at 0.2 U/mL, and 100 % (CC_100_) at 0.5 U/mL (Figure 2A down(Fig. 2)). In T-ALL cell lines CEM and MOLT-4 30 % of cell death (CC_30_) was reached at 0.4 U/mL, 50 % (CC_50_) at 0.6 U/mL, 80 % (CC_80_) at 0.8 U/mL, and 100 % (CC_100_) 1 U/mL (Figure 2(Fig. 2)). 

To further visualize the differences on cell death induced by ICRP on breast and leukemic cell lines, we next evaluated the dependence on caspases in ICRP-induced cell death. As shown in Figure 2C(Fig. 2), the percentage of ICRP-induced cell death in MCF-7 cells did not change in presence of QVD (pan caspase inhibitor), passing from 49±4.6 % in control to 49.48±8.8 % in ICRP-treated cells (p>0.05). Interestingly in 4T1 cells QVD significantly potentiated the cell death induced by ICRP, passing from 49.6±5.9 % to 59.5±7.1 % (p=0.005). On T-ALL cell lines the cell death induced by ICRP diminishes in the presence of QVD, passing in CEM from 47±5.1 % to 26.15±8.8 % (p=0.001) and in MOLT-4 from 49.5±4.9 % to 22.8±4.3 % (p=0.001). These results indicate that ICRP induces a caspase-independent cell death on breast cancer cell lines, but caspase-dependent cell death on leukemic cells.

It is known that ICRP-induced cell death relies on ROS production in solid and leukemic cancer cell lines (Lorenzo-Anota et al., 2020[21]; Martinez-Torres et al., 2018[24], 2019[22][23]; Reyes-Ruiz et al., 2021[33]). The ROS implication on ICRP-induced cell death was assessed here on breast and leukemic cell lines. First, we determined that N-Acetylcysteine (NAC) prevents ROS production by I-CRP in MCF-7, 4T1, CEM and MOLT-4 cell lines (Supplementary Figure 2). Then, in Figure 2(Fig. 2)D we show that cell death triggered by ICRP diminished from 49±4.6 % to 18.9±9 % in MCF-7 cells (p=0.0006), from 49.6±5.9 % to 15.65±11.7 % in 4T-1 cells (p=0.0104), from 47±5.1 % to 24.1±6.3 % in CEM cells (p=0.001), and from 49.5±4.9 % to 26.4±5.6 % in MOLT-4 cells (p=0.001) in presence of the ROS scavenger NAC. These data demonstrate that ICRP induces different ROS-dependent cell death modalities on breast cancer and T-ALL cell lines.

### IMMUNEPOTENT-CRP induces ROS-dependent autophagy on breast cancer and T-ALL cells

**Figure 3:**
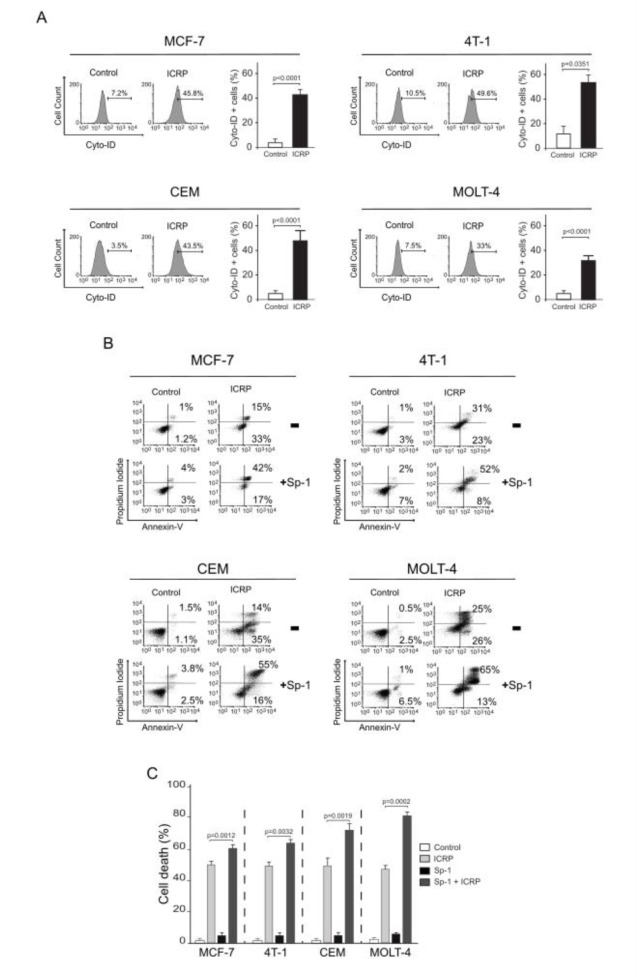
IMMUNEPOTENT-CRP induces prosurvival autophagosome formation on breast cancer and in T-ALL cell lines. A. Representative histograms (left) and quantification (right) of autophagosome formation measured by flow cytometry using Cyto-ID staining in breast cancer MCF-7 and 4T1 cells, and leukemic CEM and MOLT-4 cell lines treated with ICRP CC_50_ (24 h). B. Representative dot plots (left) of cell death analysis in MCF-7, 4T1, CEM and MOLT-4 cell lines left alone or pretreated with the autophagy inhibitor Sp-1 before ICRP treatment CC_50 _(24 h) and quantification (C). The means (± SD) of triplicates of at least three independent experiments were graphed.

It has been reported that intracellular ROS augmentation could induce autophagy, which plays an important role on cell survival (Inguscio et al., 2012[15]). Thus, we evaluated if ICRP could induce autophagy on leukemic cell lines as in breast cancer cells. In Figure 3A(Fig. 3) we can depict representative histograms (left) and quantification (right) of autophagosome formation. As shown, ICRP increased autophagosome formation in breast cancer cell lines passing from 7.5±2.6 % to 45.8±4.1 % in MCF-7 (p<0.0001) and from 10.5±11.7 % to 49.6±5.9 % in 4T1 (p=0351), and in leukemic cells from 3.5±11.7 % to 43.5±2 % in CEM (p<0.0001), and from 7.6±4.0 % to 33±9.1 % (p<0.0001) in MOLT-4 cells. Interestingly, as previously indicated for breast cancer cells, NAC was able to completely inhibit autophagosome formation in leukemic cell lines (Supplementary Figure 2), indicating that the exacerbated ROS production provoked by ICRP increases autophagosome formation.

During specific circumstances autophagy could play two principal roles, one as a cell survival strategy or cell death (Inguscio et al., 2012[15]). For this, the next step was to evaluate if autophagosome formation produced by an exacerbated ROS production could play a fundamental role on survival or death. For this, we tested cell death in presence of Sp-1, an autophagic inhibitor that effectively inhibited autophagosomes induced by ICRP in breast cancer and leukemic cell lines (Supplementary Figure 3). In Figure 3B(Fig. 3) we observe the representative dot plots obtained from cell death analyses in the four cell lines assessed, while in Figure 3C we can observe the quantification in the graph. Cell death increased significantly when autophagy was inhibited (in the presence of Sp-1). In MCF-7 cells, cell death increased from 49.6±4.6 % to 59.3±3.8 % (p=0.0012), in 4T1 cells from 49.7±5.9 % to 60.5±7.9 % (p=0.0032), from 48.1±4.1 % to 71.8±6.8 % (p=0.0019) in CEM cells and from 51±9.1 % to 78±6.0 % (p=0.0002) in MOLT-4 cells. Taken together, the increase of ROS production induced by ICRP treatment provokes ROS-dependent autophagy in breast cancer and leukemic cell lines.

### IMMUNEPOTENT-CRP induces ER stress and increases cytoplasmic Ca^2+ ^levels in breast cancer and T-ALL cells

**Figure 4:**
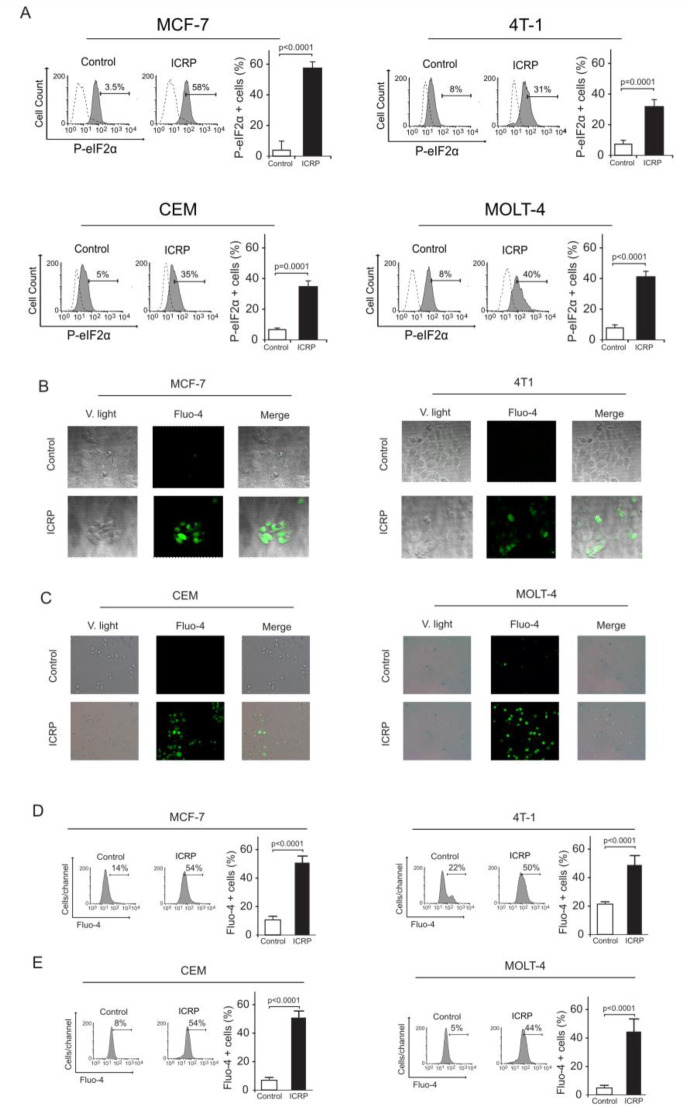
IMMUNEPOTENT-CRP induces eIF2α phosphorylation and increases in the cytoplasmic Ca^2+ ^levels. A. Representative histograms and quantification of eIF2α phosphorylation measured by flow cytometry using anti-EIF2S1 monoclonal antibody in breast cancer cell lines MCF-7 and 4T1 (left), and leukemic cells lines CEM and MOLT-4 (right). B. Confocal microscopy representation of Ca^2+ ^cytoplasmic levels measured by Fluo-4AM staining in breast cancer cell lines MCF-7 (left) and 4T1 (right) in absence (control) or presence of ICRP CC_50 _for 18 h and visualized using fluorescence microscopy (OLYMPUS X70) (40×). C. Fluorescence microscopy representation of Ca^2+ ^cytoplasmic levels measured trough Fluo-4AM staining in leukemic cell lines CEM (left) and MOLT-4 (right) in absence (control) or presence of ICRP CC_50 _for 18 h and visualized using fluorescence microscopy (OLYMPUS IX70) (40×). D-E. Representative histograms and quantification of Ca^2+ ^cytoplasmic levels assessed through Fluo-4AM staining by flow cytometry in breast cancer cell lines MCF-7 and 4T1 (above) (D), and in T-ALL cell lines CEM and MOLT-4 (down) (E) treated with ICRP CC_50 _for 18 h. Graphs represent the mean (± SD) of triplicates of at least three independent experiments.

ROS generation is strongly associated with endoplasmic reticulum alterations which lead to a stress condition in this organelle (Zeeshan et al., 2016[41]), promoting eIF2α-phosphorylation (P-eIF2α) (Rashid et al., 2015[32]). In this context, autophagy could play a crucial role during ER stress as a protective mechanism (Deegan et al., 2013[11])*.* ER stress and autophagy are two cross-talking processes (Orrenius et al., 2003[28]). As previously shown, ICRP induces ROS augmentation promoting autophagosome formation. Hence, we evaluated eIF2α phosphorylation (P-eIF2α) in breast cancer and leukemic cell lines by flow cytometry. As observed in Figure 4A(Fig. 4), ICRP causes P-eIF2α in MCF-7 (58±8.7 %), 4T1 (31±4.2 %), CEM (35±7.9 %), and MOLT-4 (40±6.5 %) cell lines, revealing, that ICRP induced P-eIF2α, one of the principal biomarkers of ER stress on breast and leukemic cell lines.

In earlier stages of ER stress conditions, intracellular alterations could induce ER-calcium (Ca^2+^) release, increasing the cytosolic Ca^2+^ concentration (Zeeshan et al., 2016[41]). The presence of autophagy and P-eIF2α suggested alterations in the ER, therefore we evaluated the increase in cytosolic calcium. Results indicated that treatment with ICRP induced an augmentation of Ca^2+^ levels in the cytoplasm in comparison with untreated cells (control) in breast cancer (MCF-7 and 4T1) (Figure 4B(Fig. 4)) and leukemic (CEM and MOLT-4) (Figure 4C(Fig. 4)) cell lines. The increase in cytosolic Ca^2+^ levels was confirmed by flow cytometry as shown in Figure 4D and 4E(Fig. 4), where we show a representative histogram (left) and quantification (right). We observed that ICRP increases cytosolic level of Ca^2+^ in breast cancer cells MCF-7 10.7±3.4 % to 54.2±4.9 % (p<0.0001) and 4T1 17.5±3.4 % to 50.8±6.0 % (p<0.0001) and in T-ALL cell lines CEM 9.8±2.2 % to 54.2±5.4 % (p<0.0001) and MOLT-4 5.6±3.1 % to 44.7±4.4 % (p<0.0001). Additionally, in all cancer cell lines (Supplementary Figure 4) the extracellular Ca^2+^ chelator (BAPTA) significantly inhibited the augmentation of cytoplasmic Ca^2+^ levels, revealing, that ICRP induces calcium alterations on cancer cell lines.

### Calcium alterations induced by IMMUNEPOTENT CRP promote mitochondrial damage, ROS production and calcium-dependent cell death on breast cancer and T-ALL cells

**Figure 5:**
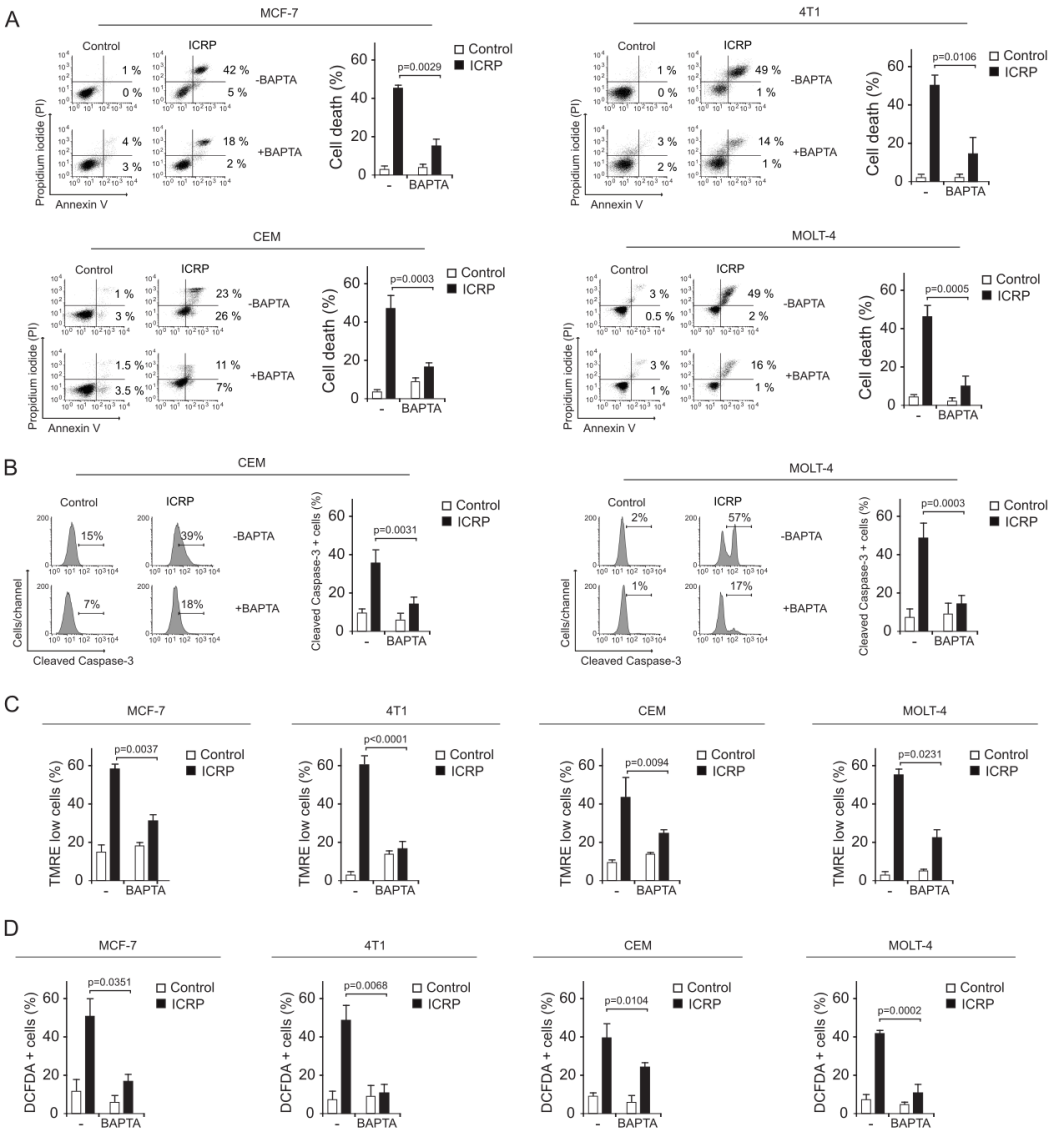
IMMUNEPOTENT CRP induces Ca^2+^-dependent cell death in tumoral and leukemic cell lines. A. Representative dot plots and quantification of cell death measured by flow cytometry through Annexin-V and PI staining in breast cancer cell lines MCF-7 and 4T1 (above), and in T-ALL cell lines CEM and MOLT-4 (down) treated with ICRP CC_50 _for 24 h in presence or absence of BAPTA. B. Representative histograms and quantification of caspase-3 cleaved by flow cytometry using FITC-DEVD-FMK staining in T-ALL cell lines CEM (left) and MOLT-4 (right) treated with ICRP CC_50 _for 24 h in presence or absence of BAPTA. C-D. Quantification of loss of mitochondrial membrane potential (C) and ROS production (D) evaluated through TMRE and DCFDA staining, respectively, by flow cytometry in breast cancer cell lines MCF-7 and 4T1, and in T-ALL cell lines CEM and MOLT-4 treated with ICRP CC_50 _for 24h in presence or absence of BAPTA. Graphs represent the mean (± SD) of triplicates of at least three independent experiments.

Cytoplasmic Ca^2+ ^overload is associated with different cell death modalities (Orrenius and Zhivotovsky, 2015[29]; Zhivotovsky and Orrenius, 2011[43]). As an increase of cytosolic Ca^2+^ levels were observed in all cancer cells after ICRP-treatment, we assessed the implication of Ca^2+^ augmentation on cell death induced by ICRP. First, cell death was evaluated in presence or absence of BAPTA. As observed in Figure 5A(Fig. 5), ICRP induced cell death in up to 50 % of cells after 24 h of treatment, and the ICRP cytotoxicity was significantly inhibited in presence of BAPTA from 47.3±4.8 % to 20.1±3.9 % in MCF-7 (p=0029), since 49.8±5.2 % to 14.2±8.7 % in 4T1 (p=0.0106), from 45.5±9.0 % to 20.9±7.0 % in CEM (p=0.0003) and in MOLT-4 from 48.9±9.3 % to 17.3±3.4 % (p=0.0005). These results indicate that ICRP induces calcium-dependent cell death on breast and leukemic cell lines.

As previously shown, ICRP treatment induces caspase-independent cell death in breast cancer cells (Martínez-Torres et al., 2020[25]; Reyes-Ruiz et al., 2021[33]), however it triggers apoptosis involving caspase-3 cleavage (a principal effector caspase) on leukemic cell lines (Lorenzo-Anota et al., 2020[21]). Thus, we tested cleaved caspase-3 on CEM, and MOLT-4 cell lines treated with ICRP. In Figure 5B(Fig. 5) we showed representative histograms (left) and quantification of cleaved caspase-3. As shown, the pre-treatment with BAPTA prevented caspase-3 cleavage on CEM (39±12.1 % to 18.37±5.4 %) (p=0.0031) and MOLT-4 (57±3.8 % to 17±3.6 %) (p=0.0003) cell lines, suggesting that calcium alteration induced by ICRP promotes caspase-3 activation that culminates in apoptosis pathway in leukemic cells.

The cytoplasmic Ca^2+ ^overload has been associated with mitochondrial alterations in different cell death pathways (Orrenius and Zhivotovsky, 2015[28]; Zhivotovsky and Orrenius, 2011[43]). Thus, the loss of mitochondrial membrane potential and ROS production in presence of BAPTA, were analyzed. ICRP treatment induced loss of mitochondrial membrane potential in all cell lines (Figure 5C(Fig. 5)) and was inhibited in presence of calcium chelator BAPTA from 59±5.4 % to 33±3.4 % (p=0.0037) in MCF-7, from 60.5±4.8 % to 18.4±2.4 % (p<0.0001) in 4T1, from 41.1±10.8 % to 22±7.2 % (p=0.0094), and from 56.2±2.6 % to 21.6±6.2 % (p=0.0231).

ICRP treatment-induced ROS production at MCF-7 (49.7±9.7 %), 4T1 (47.7±8.3 %), CEM (38.6±7.3 %), and MOLT-4 (41.6±3.5 %) also decreased in the presence of BAPTA to 19.4±6.9 %, 10.1±4.7 %, 26.2±2.7 %, and 13.6±7.1 %, respectively (Figure 5D(Fig. 5)). These data demonstrate that ICRP-mediated cell death relies on the cytoplasmic increase of Ca2+ levels, being the first event observed during the ICRP-cytotoxic pathway. Moreover, Ca^2+^ deregulation is an invariable event that occurs in the caspase-independent or dependent pathways induced by ICRP.

### Inhibition of ER-Ca^2+^ channels block ROS production and cell death induced by IMMUNEPOTENT-CRP 

**Figure 6:**
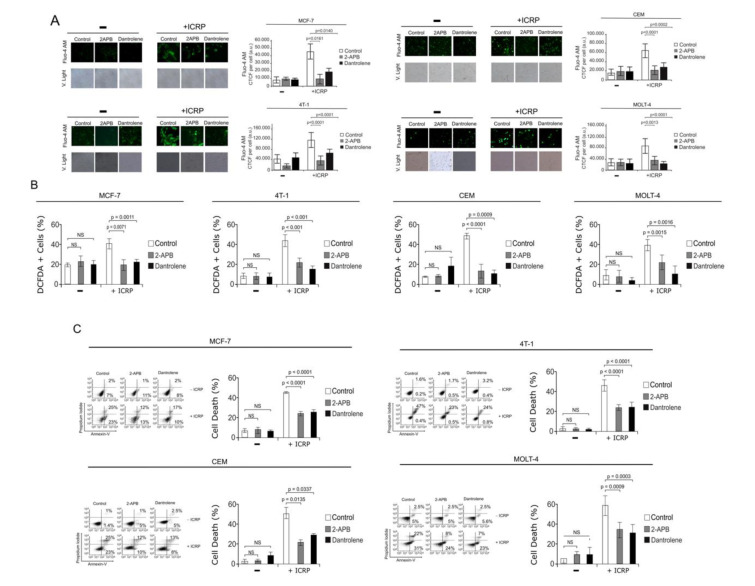
IMMUNEPOTENT-CRP induces Ca^2+^-dependent cell death in breast cancer and leukemic cell lines. A. Fluorescence microscopy representation (left) and quantification (right) of Ca^2+ ^cytoplasmic levels measured through Fluo-4AM staining in breast cancer MCF-7 and 4T1 and leukemic cell lines CEM and MOLT-4 treated with ICRP CC_50 _for 18 h and in presence or absence of 2-APB and Dantrolene, visualized using fluorescence microscopy (OLYMPUS IX70) (40×). B. Quantification of ROS production assessed by DCFDA staining by flow cytometry in breast cancer cell lines MCF-7 and 4T1, and in T-ALL cell lines CEM and MOLT-4 treated with ICRP CC_50 _for 24 h in presence or absence of 2-APB and Dantrolene. C. Quantification of cell death measured by flow cytometry through Annexin-V and PI staining in breast cancer cell lines MCF-7 and 4T1 (above), and T-ALL cell lines CEM and MOLT-4 (down) treated with ICRP CC_50 _for 24 h in presence or absence of 2-APB and Dantrolene. Graphs represent the mean (± SD) of triplicates of at least three independent experiments.

Inositol 1,4,5-trisphosphate receptors (IP_3_R) and ryanodine receptors (RyR) are the principal mediators of Ca^2+^ release from intracellular stores (Hanson et al., 2004[13]; Missiaen et al., 2001[26]; Splettstoesser et al., 2007[39]). We blocked IP_3_R and RyR, using 2-APB (Splettstoesser et al., 2007[39]) and Dantrolene (Zhao et al., 2001[42]) respectively, to evaluate their implication on the mechanism induced by ICRP. We observed by fluorescent microscopy that in presence of 2-APB and Dantrolene, cytoplasmic Ca^2+^ was diminished in MCF-7, 4T1, CEM and MOLT-4 cell lines (Figure 6A(Fig. 6)). 

ROS generation is strongly associated with Ca^2+^ leakage in ER (Zeeshan et al., 2016[41]). Thus, we then determined if the IP_3_R and RyR are involved on ROS production, the principal effectors on ICRP-cytotoxicity. In Figure 6B(Fig. 6) we can observe that ROS production induced by ICRP diminished in presence of 2-APB, the IP_3_R inhibitor or Dantrolene, the RyR inhibitor. On MCF-7 ROS production passed from 49.7±9.7 % to 24±4.2 % and 23.6±4.5 %, respectively, in 4T1 from 44.6±5.6 % to 22.1±4.6 % and 15.8±2.7 %, respectively. T-ALL cell lines had a similar outcome, in CEM cells with ROS production passed from 38.6±7.3 % to 13.7±8.1 % and 6.25±2.3 %, respectively, and in MOLT-4 cells from 41.6±3.5 % to 19.9±8.1 % and 9.9±8.4 %, respectively. Taken together, these results revealed that ER Ca^2+^ release induces ROS production in breast cancer and leukemic cells.

Finally, we assessed the implication of these ER-Ca^2+^ release channels in cell death. Our results in Figure 6C(Fig. 6) show that 2-APB diminished cell death on MCF-7 from 47.3 % to 25.3 % (p<0.0001), in 4T-1 from 48.6 % to 23.83 % (p<0.0001), in CEM from 50.9 % to 21.7 % (p=0.0135), and MOLT-4 from 52 % to 32 % (p=0.0009). Similar inhibition was observed using Dantrolene in cell death where it diminishes from 47.3 % to 27.4 % (p<0.0001) in MCF-7 cells, from 48.6 % to 24.01 % (p<0.0001) in 4T-1 cells, from 50.9 % to 25.8 % (p=0.0337) in CEM cells, and from 52 % to 30 % (p=0.0003) in MOLT-4 cells. 

## Discussion

ROS-dependence has been the principal hallmark of ICRP-mediated cell death, independently of the cancer cell type assessed to date (Martinez-Torres et al., 2018[22], 2019[22][23]; Reyes-Ruiz et al., 2021[33]). Here we show that ICRP induces calcium-dependent ROS production leading to cell death in T-ALL and breast cancer cell lines. Additionally, ICRP triggered caspase-dependent or caspase-independent cell death in different cancer cell lineages. This has been observed with other cell death inductors as gold nanoparticles or photodynamic treatments, which induce different cell death mechanisms in cancer cells, depending on the cancer cell type (Martínez-Torres et al., 2019[23]; Soriano et al., 2017[38]). These treatments commonly induce mitochondrial damage and ROS production (Perillo et al., 2020[30]), as we observed with ICRP. 

ROS-induced stress can be sensed and activate autophagy, which plays a role on stress adaptation and cell death (Chang and Zou 2020[6]; Cordani et al., 2019[8]). In this work, we show that ICRP induces ROS-dependent autophagosome formation, as an adaptive mechanism to avoid cell death. Other agents such as Salinomycin, Oxaliplatin and Irinotecan induce ROS-dependent prosurvival autophagy in breast cancer cell lines MDA-MB-231 and MCF-7 (Kim et al., 2017[18]), and gastric cancer cells (Shi et al., 2012[36]; Zhu et al., 2020[45]). The ROS-Autophagy-ER stress axis has been extensively linked, as ROS can promote ER-stress, mediating autophagy to abrogate cellular damage (Deegan et al., 2013[11]; Rashid et al., 2015[32]; Zeeshan et al., 2016[41]). ER stress can mediate ROS cascades which in turn can activate autophagy as a protective mechanism (Cao and Kaufman 2014[5]; Zhong et al., 2015[44]; Zeeshan et al., 2016[41]), explaining why ROS production induced by ICRP can lead to ER stress and autophagy on cancer cell lines. ER stress commonly involves P-eIF2α, which has also been linked to autophagy (Humeau et al., 2020[14]). In this sense, as I-CRP did in T-ALL and breast cancer cell lines, chemotherapeutics such as anthracyclines and oxaliplatin can induce P-eIF2α in U2OS and HCT 116 cancer cells (Bezu et al., 2018[4])*.*

ER Ca^2+^ release is highly associated with ER stress conditions (Cioffi, 2011[7]). It has been reported that during early stages of ER stress, oxidative stress forces Ca^2+^ out of ER (Li et al., 2009[20]). High ER-Ca^2+^ release through RyR and IP_3_R can provoke mitochondrial uptake generating ROS production, mitochondrial depolarization, and cell death (Kerkhofs et al., 2018[17])*. *Here we show that extracellular Ca^2+ ^chelation prevents the loss of mitochondrial membrane potential, ROS production, and cell death. It is well known that Ca^2+^ depletion from ER activates SOCE leading to high concentrations of Ca^2+ ^inside the cell (Orrenius et al., 2003[29]), explaining why BAPTA and ER-Ca^2+^ receptor inhibitors could decrease intracellular calcium augmentation. Moreover, we show that the inhibition of ER-calcium receptors (RyR and IP_3_R) inhibits ROS production and death. Our results indicate that the increase of intracellular Ca^2+^ levels induced by ICRP is the first step on both cell death modalities described so far on breast and T-ALL cell lines. 

These results are similar to the cell death pathways described with other treatments. For instance, menadione induces ER-Ca^2+^ release, accompanied by mitochondrial Ca^2+^ elevation, mitochondrial depolarization, and mitochondrial permeability transition pore (mPTP) opening, leading to cell death in pancreatic tumor cells (Baumgartner et al., 2009[3]). Ceramide-induced cell death in HeLa cells involves ER-Ca^2+^ release and mitochondrial Ca^2+^ increase, accompanied by marked alterations in mitochondria morphology (Pinton et al., 2001[31]). Also, cisplatin increased cytoplasmic and mitochondrial Ca^2+^ levels in HeLa cells, which further triggered mitochondrial-mediated and ER stress-associated cell death pathways, moreover, the inhibition of IP_3_R decreased calcium release from the ER and inhibited cisplatin-induced cell death (Shen et al., 2016[35]). In this context, an IP_3_ receptor antagonist, 2-APB attenuates cisplatin induced Ca2^+^-influx in HeLa-S3 cells and prevents activation of calpain and induction of apoptosis (Splettstoesser et al., 2007[39]).

Overall, our results revealed that IMMUNEPOTENT CRP triggers endoplasmic reticulum stress accompanied by the increase of intracellular Ca^2+^ levels leading to mitochondrial damage and ROS production provoking regulated cell death on cancer cell lines. This work opens new possibilities to evaluate the cytotoxicity of ICRP in combination with other types of cell death inductors, and the immunogenicity of the cell death in hematological malignances and other solid cancers.

## Notes

Helen Y. Lorenzo-Anota and Alejandra Reyes-Ruiz contributed equally as first author.

Ana Carolina Martínez-Torres and Cristina Rodríguez-Padilla contributed equally as last author.

## Acknowledgements

HYLA, ARR, KMCR, RMR, and APUP thank CONACyT for scholarship. We thank the Laboratorio de Inmunología y Virología from Facultad de Ciencias Biológicas of the Universidad Autónoma de Nuevo Léon for the facilities provided to achieve this work. 

## Supplementary Material

Supplementary information

## Figures and Tables

**Figure 1:**
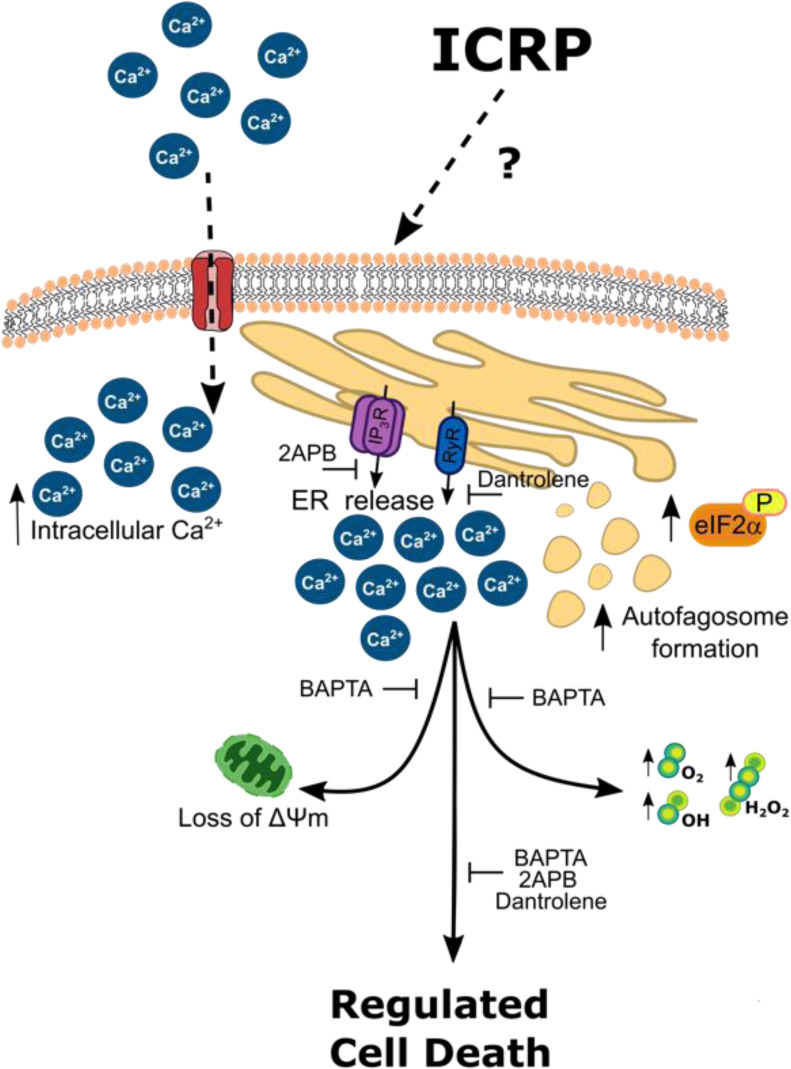
Graphical abstract
