# Supplementary material for: IMMUNEPOTENT CRP increases intracellular calcium through ER-calcium channels, leading to ROS production and cell death in breast cancer and leukemic cell lines
Source: EXCLI J. 2023 Mar 16;22:352–66. doi: 10.17179/excli2022-5568 (PMC10201010; doi:10.17179/excli2022-5568)
Supplement: Supplementary information [file EXCLI-22-352-s-001.pdf]

EXCLI Journal 2023;22:352-366 – ISSN 1611-2156
Received: December 13, 2022, accepted: February 08, 2023, published: March 16, 2023

Supplementary information to:

Original article:

IMMUNEPOTENT CRP INCREASES INTRACELLULAR CALCIUM THROUGH ER-CALCIUM CHANNELS, LEADING TO ROS PRODUCTION AND CELL DEATH IN BREAST CANCER AND LEUKEMIC CELL LINES

Helen Y. Lorenzo-Anota[1,2,+], Alejandra Reyes-Ruiz[1,+], Kenny M. Calvillo-Rodríguez[1], Rodolfo Mendoza-Reveles[1], Andrea P. Urdaneta-Peinado[1], Karla M. Alvarez-Valadez[1], Ana Carolina Martínez-Torres[1^*], Cristina Rodríguez-Padilla[1,3,^]

1 Laboratorio de Inmunología y Virología, Facultad de Ciencias Biológicas, Universidad Autónoma de Nuevo León, San Nicolás de los Garza, México
2 Tecnológico de Monterrey, The Institute for Obesity Research, Monterrey, México
3 LONGEVEDEN S.A. de C.V.

+ Co-first authors
^ Co-senior authors

* **Corresponding author:** Ana Carolina Martínez-Torres. Laboratorio de Inmunología y Virología, Facultad de Ciencias Biológicas, Universidad Autónoma de Nuevo León, San Nicolás de los Garza, México. E-mail: ana.martinezto@uanl.edu.mx

https://dx.doi.org/10.17179/excli2022-5568

This is an Open Access article distributed under the terms of the Creative Commons Attribution License (https://creativecommons.org/licenses/by/4.0/).

EXCLI Journal 2023;22:352-366 – ISSN 1611-2156
Received: December 13, 2022, accepted: February 08, 2023, published: March 16, 2023

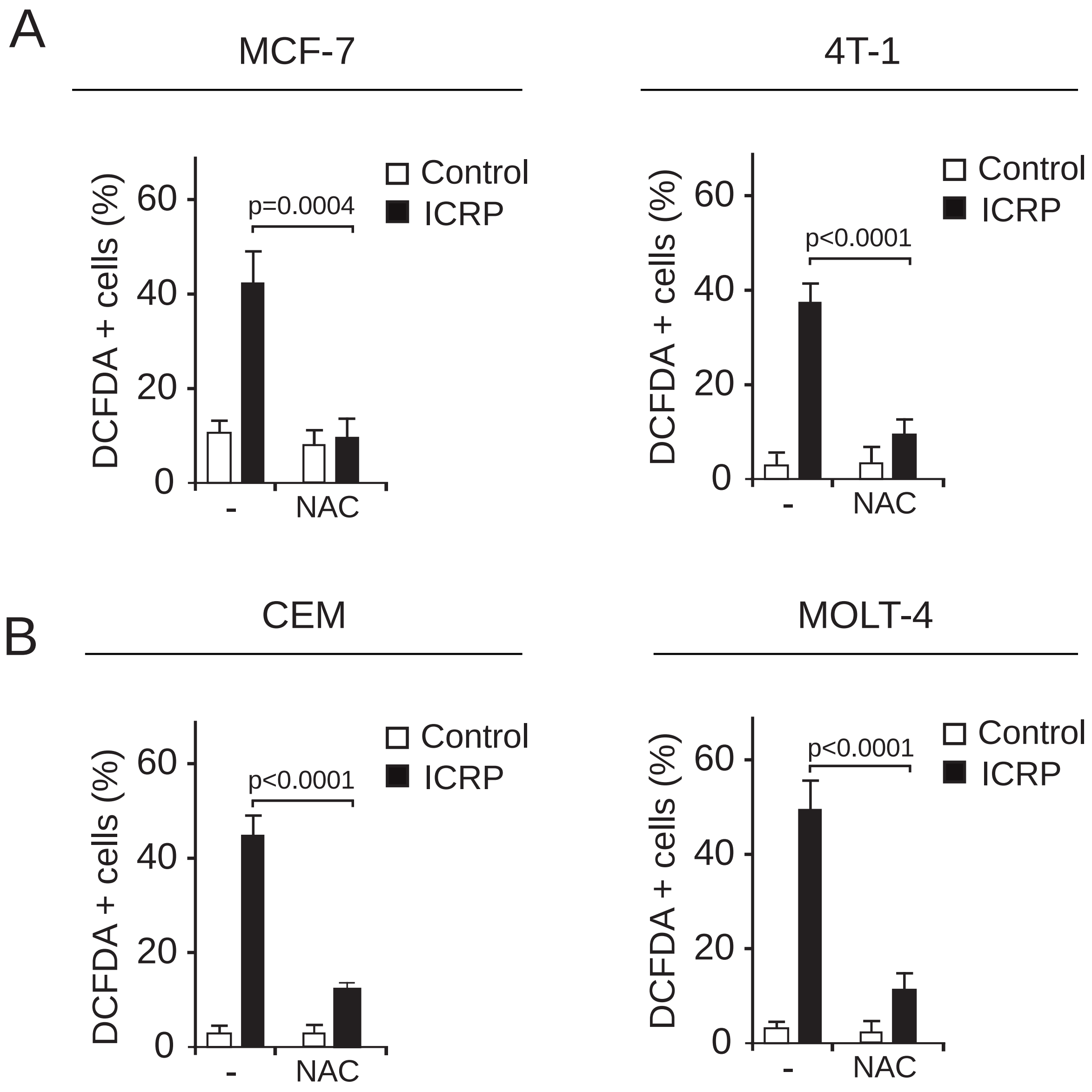

Supplementary Figure 1: NAC inhibits ROS production in breast cancer and T-ALL cell lines. Quantification of ROS production using DCFDA staining by flow cytometry **A.** in breast cancer cell lines MCF-7 and 4T1, and **B.** in T-ALL cell lines CEM and MOLT-4 treated with ICRP CC_{50} for 24 h in presence or absence of NAC. Graphs represent the mean (± SD) of triplicates of at least three independent experiments.

EXCLI Journal 2023;22:352-366 – ISSN 1611-2156
Received: December 13, 2022, accepted: February 08, 2023, published: March 16, 2023

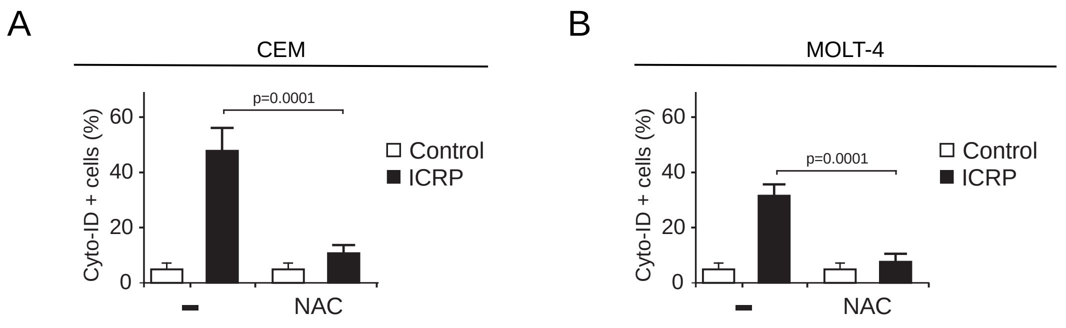

Supplementary Figure 2: NAC inhibits autophagosome formation in T-ALL cell lines. Quantification of autophagosomes using Cyto-ID staining by flow cytometry **A.** CEM and **B.** MOLT-4 cell lines treated with ICRP CC_{50} for 24 h in presence or absence of NAC. Graphs represent the mean (± SD) of triplicates of at least three independent experiments.

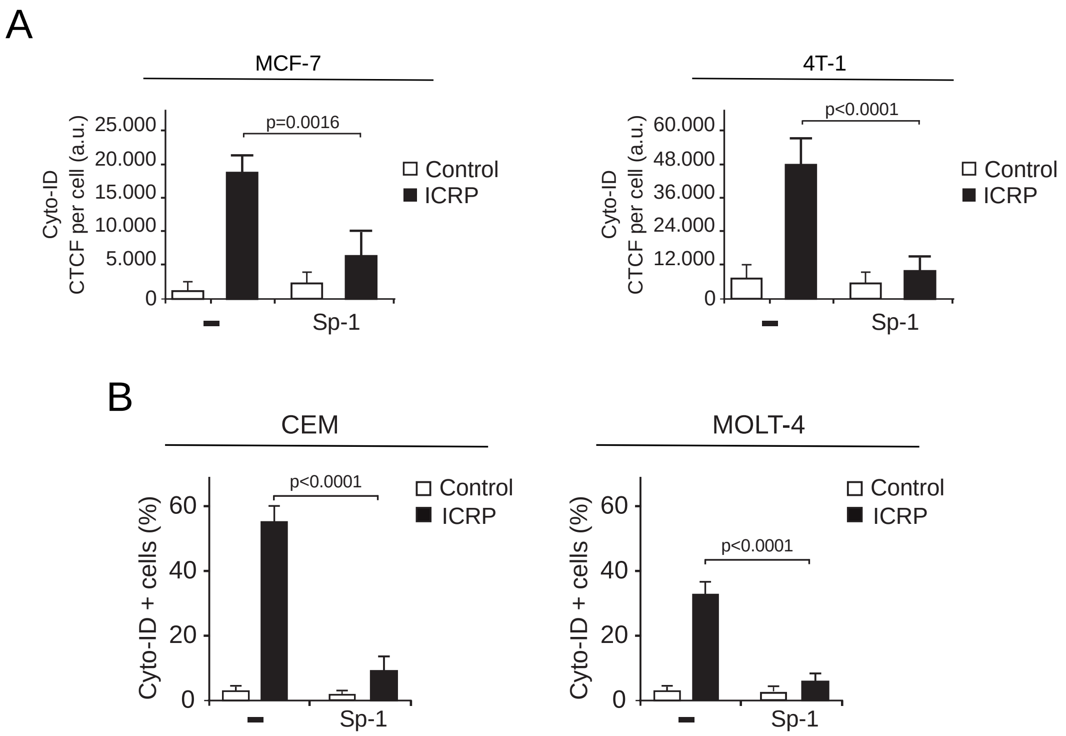

Supplementary Figure 3: Spautin-1 inhibits autophagosome formation in breast cancer and T-ALL cell lines. A. Corrected total cell fluorescence (CTCF) of Cyto-ID staining shown in arbitrary units (a.u.) in MCF-7 and 4T1 cells left untreated (control) or treated with ICRP CC_{50} for 24 h without co-treatment (-) or co-treated with Spautin-1 (Sp-1). The means (± SD) of triplicates of at least three independent experiments were graphed. **B.** Quantification of autophagosomes using Cyto-ID staining by flow cytometry in CEM and MOLT-4 cell lines treated with ICRP CC_{50} for 24 h in presence or absence of Sp-1. Graphs represent the mean (± SD) of triplicates of at least three independent experiments.

EXCLI Journal 2023;22:352-366 – ISSN 1611-2156
Received: December 13, 2022, accepted: February 08, 2023, published: March 16, 2023

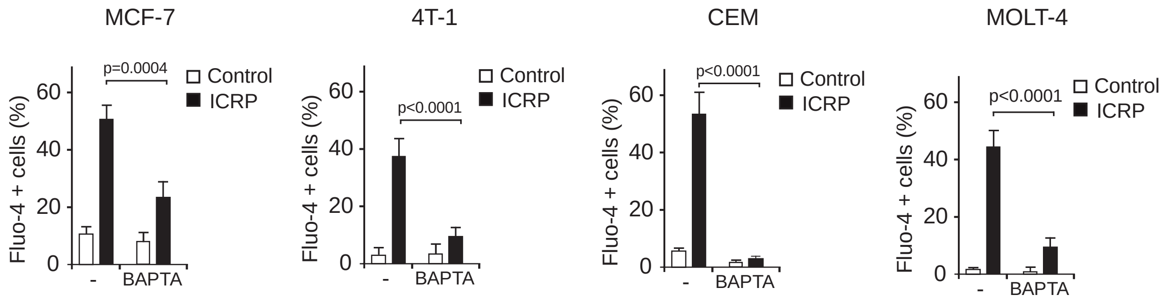

Supplementary Figure 4: BAPTA inhibits intracellular Ca^{2+} augmentation in breast cancer and T-ALL cell lines. Quantification of cytoplasmic Ca^{2+} levels assessed trough Fluo-4AM staining by flow cytometry in breast cancer cell lines MCF-7 and 4T1 and in T-ALL cell lines CEM and MOLT-4. Cells were left untreated (control) or treated with ICRP CC_{50} for 18 h, in the absence or presence of BAPTA. Graphs represent the mean (± SD) of triplicates of at least three independent experiments.
